# Information Is Selection—A Review of Basics Shows Substantial Potential for Improvement of Digital Information Representation

**DOI:** 10.3390/ijerph17082975

**Published:** 2020-04-24

**Authors:** Wolfgang Orthuber

**Affiliations:** Department of Orthodontics, UKSH, Kiel University, 24105 Kiel, Germany; orthuber@kfo-zmk.uni-kiel.de

**Keywords:** big data, efficiency, similarity search, information, selection, online definition, adapted domain, metric space, domain vector, domain space

## Abstract

Any piece of information is a selection from a set of possibilities. In this paper, this set is called a “domain”. Digital information consists of number sequences, which are selections from a domain. At present, these number sequences are defined contextually in a very variable way, which impairs their comparability. Therefore, global uniformly defined “domain vectors” (DVs), with a structure containing a “Uniform Locator” (“UL”), referred to as “UL plus number sequence”, are proposed. The “UL” is an efficient global pointer to the uniform online definition of the subsequent number sequence. DVs are globally defined, identified, comparable, and searchable by criteria which users can define online. In medicine, for example, patients, doctors, and medical specialists can define DVs online and can, therefore, form global criteria which are important for certain diagnoses. This allows for the immediate generation of precise diagnostic specific statistics of “similar medical cases”, in order to discern the best therapy. The introduction of a compact DV data structure may substantially improve the digital representation of medical information.

## 1. Introduction

Information is typically represented in a very variable manner, such that its comparison is often made difficult or even impossible. This is a very important shortcoming in the case of medical information, with direct consequences for therapy. Therefore, one aim of this article is to recall the underlying theoretical and technical details of information. Digitally, information is a number sequence which is always a selection from a common ordered set of possibilities (this set is called a “domain” herein). In this paper, it is explained in detail that this domain crucially determines the digital representation of information and its comparability. Furthermore, it is also shown that the internet provides an efficient possibility for the long-term improvement of the current situation, through the online definition of adapted domains and of number sequences (which select from the domains and which are called “domain vectors”). These form the basis of a new type of language-independent medical information, which is globally comparable and searchable by means of user-defined criteria (e.g., defined by medical specialists) which, therefore, makes it interesting and relevant to users. To construct the infrastructure for this approach, it is recommended that an attractive internet presence for the online definition of adapted domains by users is created.

## 2. Definition of Information

In terms of “information”, the exact and complete concept is meant here. This should not be exchanged with “information quantity”, which can be measured by counting bits, and which is only one property of information. There is a large amount of literature about information; however, imprecise and unclear concepts have been used for the definition of the exact term “information”. For the quantification of similarity and for the general comparison of information, a clear, precise, and natural approach is necessary. For this, it should be recalled that information is selection. It is well-known that any piece of digital information is a bit sequence and, therefore, a selection. Information, in general, as a result of any physical experiment, is also a selection (from a set of possible results; see, e.g., page 6 of Dirac’s book [1]). The approach proposed here consequently begins with this definition:

“Information is selection from a domain.”(1)

Here, “domain” denotes an ordered set of possibilities, which are common between the sender and receiver of the information. Information is always associated with a domain, which, in turn, is the domain of the information. The sender and receiver must both know the domain; for example, they must have a common vocabulary. Then, information is processed and transported digitally as a selection from the domain, as a number sequence. The domain of information crucially determines its digital representation. Therefore, information is fully defined by its domain and its selection from the domain [2,3,4].

It is important that (1) is a fundamental principle which is generally valid, even in elementary physics, and more research concerning this is recommended. For example, a common elementary charge and the derived common set (domain) of multiples of this elementary charge are preconditions for any electronic communication.

## 3. Global Definition of Information

Digital information consists of number sequences which are defined, by context, in a variable way. This can be improved by globally defining the domains of digital information (respective number sequences) in a uniform machine-readable way on the internet (i.e., as uniform online definitions of an ordered set). Let “Uniform Locator” (“UL”) denote an efficient link to the online definition of the domain of the subsequent number sequence. Then, the data structure can be used to transport any globally defined digital information. This is called a “domain vector” (“DV”). The online definition of the domain is the global predefinition of information [2]. As the DV contains the UL of this predefinition of the domain and the number sequence, which selects in this domain, the DV represents globally defined information.

UL plus number sequence(2)

### 3.1. Literature Research

Usually, information is implicitly regarded as a selection from a set of possibilities (i.e., a domain). However, the global and uniform definition of this set is not focused. For an extensive literature review in this paper, Google Scholar [5] was used, with “Information” and “Definition” used as search terms, without any restrictions on publishing dates. A more restrictive search was also done, and other search engines were also used. Except for the author’s own publications (e.g., [2,3,4]), there were no relevant studies which focused on the definition of information using a global definition of a common set of possibilities or domain!

### 3.2. Format of the Domain Vector (DV)

The DV is introduced in more detail in [2]. Here, a short description of the binary format is provided, in order to clarify the efficiency of the approach:

1. The UL has a similar function to a link (i.e., a URL resp. “Uniform Resource Locator”) [6], but allows for maximal efficiency. It is a number sequence and typically has a hierarchical structure with a predefined meaning, where the first number represents the count of the subsequent numbers of the UL and the second number points to a global table of conventional internet addresses of online presences, where users can define DVs online in a standardized way. Subsequent numbers in the UL can provide detailed addresses within the chosen online presence;
2. Numbers in the UL are self-elongating positive integers, starting with a half byte or byte, as shown in Figure 4 of [2];
3. The number sequence after the UL is completely defined in the online definition of the online address given by the UL. This is also a metric (i.e., a distance function; see Section 4.4) for a similarity comparison of DVs when the UL is provided and the online definition is expandable. Necessary explanations and definitions are, at least, given in English, but should be language-independent, such that translation into other languages (i.e., multilingual definitions) is possible;
4. Nesting and a posteriori combinations of DVs are possible and often efficient (e.g., date, time, and location, along with a sequence of measurement results);
5. The binary format of the DV can be converted into a text-compatible form using, for example, the Base64 Data Encoding specification (RFC 4648) [7]. After this, it can be integrated into currently recommended approaches (e.g., into the FHIR resp. “Fast Health Interoperability Resources" standard [8]) as an extension [9].

## 4. Comparison of Information

In general, information is only comparable if its domain is the same. Otherwise, the comparison and interpretation of information becomes imprecise or even impossible. Therefore, in this Section, important exemplary domains will be discussed. Then (in Section 4.4), preconditions for comparability will be defined exactly.

### 4.1. Domain of Information: “Language Vocabulary”

In the case of language-based information, the domain is “language vocabulary” (i.e., a set of commonly known words and phrases, including the special terms, of a certain language). There should be a common language, but, even in this case, the domain “language vocabulary” is not exactly the same for all speakers. This can cause misunderstandings. For example, as a comment on the weather, Alice may say “It is cold” when, at the same temperature, Bob might say “It is not cold”, because the word “cold”, as an element of the domain “language vocabulary” for Bob (who may wear warmer clothes) has another definition than for Alice. A further deep problem is caused by combinatorial complexity and redundancy. Multiple phrases are possible in the same situation. For example, in this situation, Alice may also say “I’m freezing”.

### 4.2. Translation of Original Information into Digital Representation using the Domain “Language Vocabulary”

Let us denote, by original information (“ORGINFO”), certain relevant original (language-independent) information that should be transported digitally as digital information (“DIGINFO”). In the case of typical language-based communication, ORGINFO is coded and transported by combinations of the domain “language vocabulary”. In the case of non-trivial ORGINFO, these combinations of words are long. As such, the coding (or representation) of ORGINFO by a free language is done in a non-reproducible way, and there is large variability in the resulting language-based digital representation, DIGINFO.

**Figure 1:**
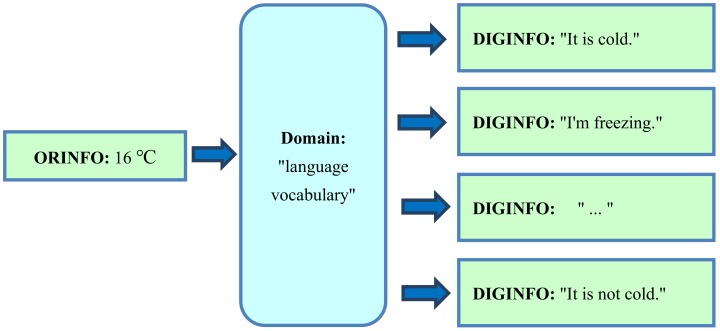
Even if the domain “language vocabulary” of the same language is used, the original information (ORGINFO), e.g., “The temperature is 16 °C”, can be translated in several ways into its digital representation, digital information (DIGINFO). The results are imprecise.

For an illustration of the principle, we first start with the abovementioned simple weather commentary example, assuming that the original situation ORGINFO means “The temperature is 16 °C”, which caused Alice to say “It is cold”. Using “language vocabulary” as the domain, ORGINFO can be represented as DIGINFO in several ways, as Alice could also say “I’m freezing” or Bob could even say “It is not cold”. In every case, Alice and Bob think that they translated ORGINFO correctly into language, but the resultant DIGINFO is so imprecise that it can even look contradictory (Figure 1).

**Figure 2:**
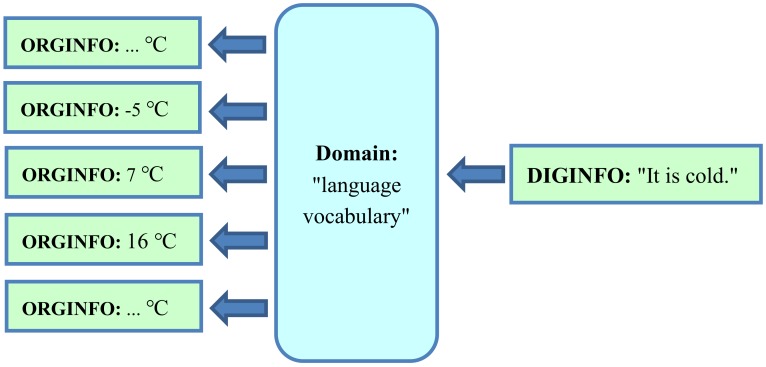
Using the domain “language vocabulary”, an exemplary text search of “It is cold” finds textual representations of very different original temperatures (ORGINFO).

Conversely, when searching for ORGINFO using the domain “language vocabulary”, several terms can be entered. The precise term “16 degrees Celsius” is too seldom used in conventional texts to be representative of ORGINFO. Moreover, similar situations are also interesting—for example, the precise term “15 degrees Celsius”, as a measurement result of temperature, in all languages. As a text search of all possibly interesting precise terms is not practicable, the term “temperature” can be used to represent the imprecise term “It is cold”, as shown in Figure 2. The search results represent very different original temperatures. More useful results are possible by searching for a longer, more specific text which represents additional features—for example, by searching for the combination “cold indoor temperature”. Some search results may already contain helpful information. Therefore, a text search is far better than nothing. Nevertheless, basic problems (e.g., incompleteness, overlapping, redundancy, imprecision) related to forward (Figure 1) and backward (Figure 2) translations of original information (ORGINFO) in the domain “language vocabulary” remain.

Completely different combinations of words or phrases (elements of the domain “language vocabulary”), as shown on the right side of Figure 1, can have the same intended meaning, as shown on the left side of Figure 1. In the case of a text search, the results may be imprecise because the meaning of the same text, as shown on the right side of Figure 2, is imprecise and corresponds to many variants of ORGINFO, as shown on the left side of Figure 2. This imprecision results from the use of the domain “language vocabulary”, which should be manageable and easily understandable.

To describe everything feasible using this domain, there is freedom in combining its elements (words and phrases). However, this leads to overlapping of meaning. The same thing can be described in several ways (i.e., by several different combinations of words). Therefore, a text search of a certain sequence yields only a part of all locations with this meaning. As the number of possible sequences increases exponentially with the count of words in the sequence, the probability of finding a certain meaning with a single word sequence decreases exponentially with the number of words in it. Thus, if more than a few words are necessary to obtain a certain meaning, the probability of finding the most interesting locations with this meaning using a text search becomes very small. Therefore, text searches are practicable only for short sequences of words.

However, in the case of professional communication (e.g., in medicine), communicated information is usually nested and non-trivial. This means that a few words are not sufficient to describe a certain situation. An additional introduction is necessary, which is too long to be searchable using a text search. As searchability and comparability of non-trivial and nested information is important, a solution is necessary.

### 4.3. Domain of Information: Adapted to the Topic

For a precise comparison and search of ORGINFO, a solution that is less variable and more reproducible than using “language vocabulary” as the domain (see Section 4.2) of DIGINFO is desirable. This is possible through the use of a topic-specific “adapted domain”, which is defined online, such that there is full reproducibility in both directions—that is, it forms a bijection (a one-to-one correspondence) between every variant in ORGINFO and their digital representations in DIGINFO.

As it is impossible to bijectively represent “all information” (i.e., “all features”) of reality digitally, the restriction to relevant features (i.e., sub-areas of information) is necessary. This is possible because ORGINFO is communicated within a certain topic—that is, it should only represent features which are relevant within the chosen topic. Thus, for the adaptation of the domain of ORGINFO to this topic, the following questions are (repeatedly) asked:

(a) Which (additional) independent feature (parameter) is relevant within the chosen topic? If an appropriate quantification of this feature is available online, reuse it; otherwise, ask:
(b) Which variants of the feature are possible? Quantify the feature, order its variants, and define a bijection to the numeric values of a parameter with the corresponding order.

For (a), relevant independent features are repeatedly searched. Every feature has variants which are selected (represented) by ORGINFO. If these are naturally ordered (e.g., have a quantitative magnitude), this order is taken; otherwise, a useful order is introduced. If the resulting order is multidimensional, every dimension can be regarded as an independent feature with a one-dimensional order.

After this, every resulting feature has a one-dimensional set of variants, such that every variant of every feature is bijectively represented (i.e., digitally selected) by a single number. Thus, the feature is quantified. If “N” denotes the count of all features, then the selection of the variants of all features is done digitally using N numbers (i.e., by an N-dimensional vector). The conversion of ORGINFO to this digital representation DIGINFO is a bijection into an N-dimensional vector space (i.e., the digital domain of DIGINFO) from the to the topic-adapted domain of ORGINFO. Due to this bijection, the domains of ORGINFO and DIGINFO can be treated as equivalent. This substantially simplifies our considerations in the case of adapted domains. 

**Figure 3:**
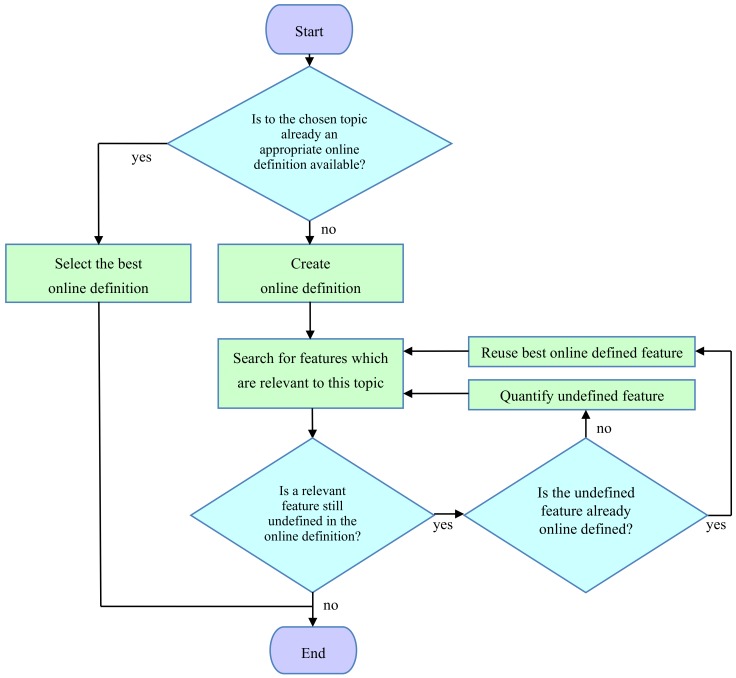
Online definition of an adapted domain.

Within the adapted domain, the relevant features of the original information are represented by numbers. Therefore, the definition of an adapted domain can be regarded as the definition of the number sequence, DIGINFO, which represents certain relevant features within the chosen topic. Adapted domains can be defined online (as described in Section 3). It is important that online definitions are globally available. To avoid redundancy, appropriate online definitions for this topic should be first searched and used before a new definition is defined. If relevant features are still undefined, their new online definition is appropriate. Figure 3 shows a flowchart of the online definition of an adapted domain.

Consider this process applied to the weather commentary example of Section 4.1, where we assume that no appropriate online definition of the topic “weather” is available. In this case, the generation of a new definition is appropriate. According to Figure 3 and Section 4.3 (a), independent relevant features within the topic “weather” are searched. There are many such features, such as atmospheric temperature, barometric pressure, relative humidity, and so on. In this example, only the feature “atmospheric temperature” is necessary. If an appropriate online definition is available, it is used; otherwise, such a definition is created. For this, the feature is quantified. In this example, the original information (ORGINFO) “atmospheric temperature” already has the internationally given ordered property T °C. Therefore, simply the letter T (which represents multiples of °C) is taken as the digital information (DIGINFO). According to Section 4.3 (a), all interesting variants of this feature are ordered to obtain a one-to-one correspondence (bijection) with the number T.

**Figure 4:**
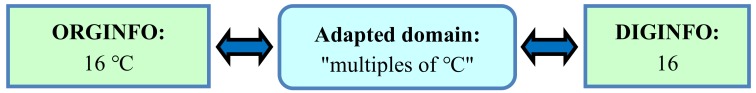
The original information (ORGINFO) “The temperature is 16 °C” is translated bijectively to its digital representation, DIGINFO. It is identified by the “Uniform Locator” (“UL”), which, according to (2), is an efficient global pointer to the online definition of the adapted domain. Due to the use of the adapted domain “multiples of °C”, there is a one-to-one correspondence of every variant of ORGINFO to its digital representation, DIGINFO.

This process is illustrated in Figure 4. The original information “The temperature is 16 °C” is represented by the single number “16”. Despite this shortness, there is a clear one-to-one correspondence between every possible variant of ORGINFO to its digital representation, DIGINFO. In contrast, Figure 1 and Figure 2 show how ambiguity and imprecision occurs, in the case of free language, due to the use of the domain “language vocabulary”.

As shown above for the feature “atmospheric temperature”, definitions of further features such as “barometric pressure”, “relative humidity”, and so on can be appended to the online definition of “weather”. This increases its dimensionality and the maximal length of the number sequence DIGINFO. If the value of a certain number is not available, it can be represented, for example, by a short placeholder in DIGINFO.

### 4.4. Comparability of Information

Let DV1, DV2, and DV3 represent variants of digital information which are elements of the same domain D (e.g., domain vectors, as defined in Section 3 with the same UL). This is the first precondition for comparability. A further precondition is a non-negative distance function (i.e., metric)

F: D × D → [0, ∞) which fulfills (3)

F(DV1, DV2) ≥ 0,
F(DV1, DV2) = 0 if and only if DV1 = DV2,
F(DV1, DV2) + F(DV2, DV3) ≥ F(DV1, DV3), and
F(DV1, DV2) = F(DV2, DV1).

A domain D with such a metric F is called a “metric space“ in the literature [10]. A metric space with domain vectors (2) as elements is called a “Domain Space” [2,3,4].

The definability of the metric F provides clear preconditions (3). for the comparability of information. The digital representation of information (DIGINFO) is always represented by a finite count of numbers (N), which can be seen as a vector in an N-dimensional vector space. There are many possibilities to define the metric F on such a vector space; the Manhattan and Euclidean metrics are well-known examples [10]. Therefore, the digital representation, DIGINFO, is always comparable. The decisive question is: is the original information (ORGINFO) comparable?

For example, there are severe difficulties in the case of the domain “language vocabulary”. According to Figure 1, the phrases “It is cold” and “I’m freezing” (as DIGINFO) can both represent the same original information (ORGINFO); however, these phrases can obviously also represent different original information. In the first case, F (“It is cold”, “I’m freezing”) is zero, but in the second case, F (“It is cold”, “I’m freezing”) is non-zero. Thus, if the domain “language vocabulary” is used, it is impossible to appropriately define F for the reliable comparison of original information (ORGINFO).

However, if an adapted domain is used, there is a bijection between the original information (ORGINFO) and its digital representation, DIGINFO (according to Section 4.3). This completely changes the situation. The definition of F on DIGINFO is directly applicable to ORGINFO (i.e., in the case of an adapted domain, the original information (ORGINFO) is comparable). For its automatic comparison, F can be used on the digital representation, DIGINFO. This is also important for similarity searches.

It is also plausible to consider the comparability of medical information before the application of Artificial Intelligence (AI) algorithms [11]; otherwise, the AI algorithm may “learn” from the wrong (i.e., non-bijective representation and, therefore, non-natural) domain of information, with unpredictable side effects.

### 4.5. Domains of Information in Databases

There are already many databases which work with “locally defined adapted domains”. In particular, if they contain quantitative measurable data, there is often already a bijection between ORGINFO and DIGINFO. For the global comparability of information, however, a global definition of the domain is also important. Therefore, according to Section 4.3 every “adapted domain” is defined online and, thus, is globally valid (Section 3). Existing databases could provide retroactive online definitions for the domains of their data, in order to ensure the global comparability of their data.

## 5. Search of Information

### 5.1. Text Search of Information

In the case of a text search, the domain is “language vocabulary”. As shown in Section 4.2, there is no bijection between the original information (ORGINFO) and the digital representation, DIGINFO, in this case and, thus, the comparability of the original information is limited or lost. Thus, as a matter of principle, the value of a text search is limited.

Special ontologies have been developed to obtain a better adaptation to applications, such as in medicine (e.g., ICD [12,13] and SNOMED CT [14,15,16]). Such ontologies can be seen as discrete domains. If they are (without legal restrictions) freely available [2], these can serve as starting points for the online definition of diagnosis-specific adapted domains, which are suitable for decisional support (see Section 5.6).

### 5.2. Search of Information in Databases

Conditional and similarity searches are, at present, typical applications in databases [17]. If such databases provide online definitions of the domains of their data (Section 3), they can make these data globally comparable and accessible for global searches.

### 5.3. Search of Information in General

General search commands define sorting criteria and additional conditions for the search result. To transfer these criteria and conditions to original information, a bijection from the digital representation, DIGINFO, to the original information (ORGINFO) is necessary.

In the case of a similarity search, a distance function is additionally necessary. The next Section explains this in detail.

### 5.4. Similarity Search of Information

Similarity searches have been well analyzed in the literature [18,19,20,21,22], as well as for medical databases [23,24,25,26]. In a similarity search, certain searched information is provided, and it is required that the most similar digital representations are listed first in the search results. This means that the searched information is compared with every occurrence that contains possibly interesting digital information (DIGINFO) using an algorithm, which provides, as a result, a number which reproducibly shows the rank of the DIGINFO in the search result.

This is simply the metric F, which was introduced in Section 4.4 (3). As not only the similarity search of a certain digital number sequence, or DIGINFO, but also the similarity search of original information is desirable, an adapted domain is necessary, such that there is a bijection from ORGINFO to DIGINFO. Obtaining original information search results (SEARCHED_ORGINFO) is made possible by using their digital representation, the digital information search results (SEARCHED_DIGINFO): the smaller the value of DISTANCE:= F(SEARCHED_DIGINFO, DIGINFO) is, the higher the result of DIGINFO and also ORGINFO will be in the search results.

This principle was used in our online prototype [27], which was programmed years ago, and which can be used not only for the definition of number sequences (vectors), but also for the quick definition of distance functions and for similarity searches.

**Figure 5:**
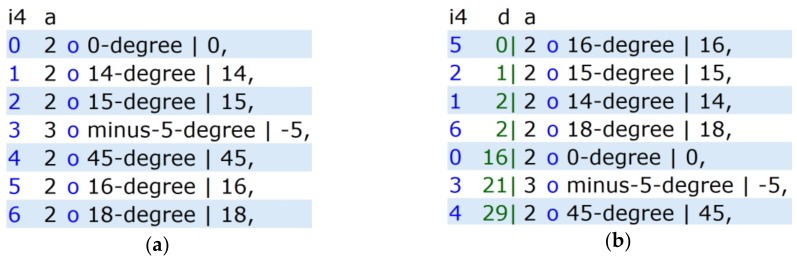
(**a**) shows a few simple “temperature” samples entered into the search prototype [27], and (**b**) shows the results of a similarity search for “16”. The most similar samples are listed first—that is, the rank (respective “similarity”) in the search result is higher when the distance is smaller. The distance is shown in column “d”, which is equivalent to the Manhattan distance F = |DIGINFO-16|. Columns: i4 = index in database; a = access count; d = |DIGINFO-16|; last column = DIGINFO.

For the example in Figure 4 (“atmospheric temperature”), the Manhattan distance (i.e., the sum of absolute differences between the values of every dimension) can be used as the distance function F. Figure 5a shows a few “temperature” samples that were entered into our search prototype [27] and Figure 5b shows the results after a similarity search for “16”. The rank (i.e., the “similarity”) of a sample in the search result is higher when the distance is smaller.

Table 1 clarifies the search results in Figure 5b and the importance of the bijection between DIGINFO and ORGINFO, which is a consequence of using the adapted domain. This is a precondition for such a precise similarity search for ORGINFO = 16 °C. In the case of the domain “language vocabulary” (text search), this is out of range, because only searching for strings as DIGINFO (e.g., “cold”) is possible. These are only loosely connected with the original information (ORGINFO).

### 5.5. User-Defined Global Similarity Search of Information

Now, we have a theoretical basis for conducting similarity searches on original information (ORGINFO). To obtain a bijection with its digital representation DIGINFO, the first step is the definition of a topic-specific adapted domain of ORGINFO (as shown in Figure 3). As described in Section 4.3, we repeatedly carry out the following two steps:

(a) Ask for relevant features within the chosen topic;
(b) Quantify them, reusing already existing online definitions.

For this, expert knowledge in the chosen field is necessary. Therefore, it is important that the users—especially experts in a certain topic—can define terms in this topic in the adapted domain with the topic-specific relevant features that they want as search criteria. The use of relevant features as criteria for similarity searches has been, up to now, a typical application in databases [28,29,30,31,32,33,34,35,36,37,38]. This restriction, however, is not necessary. After standardized online definition and global identification by UL within a domain vector (2), such features (definable by users) become globally searchable [2,3,4]. Nevertheless, this important possibility for information retrieval has not yet been realized (see Section 6.3).

### 5.6. Medical Example

Our first example, using the topic “weather” (Section 4.3) with the feature “atmospheric temperature” was introduced above. In this case, quantification is simple, because “temperature” is a well-known simple measurement and its one-dimensional representation by a single number is sufficient. More measurements, such as barometric pressure and relative humidity, can be defined in the same way and appended as further dimensions to the definition of the adapted domain “weather”. Thus, the numeric representation changes from one-dimensional to multidimensional and the definition requires more work; however, the steps are not more difficult (as long as the quantification is obvious).

**Figure 6:**
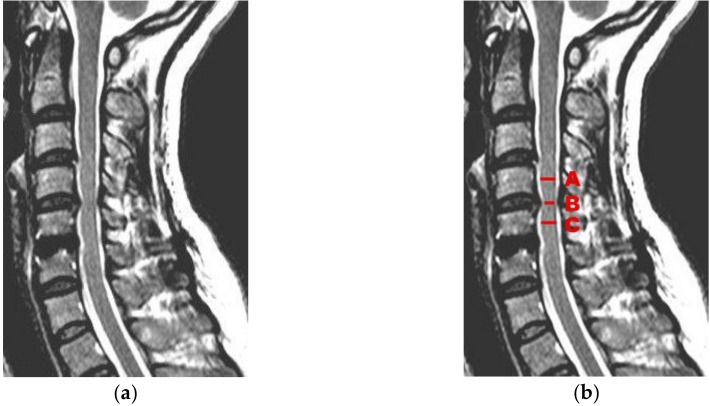
(**a**) MRI of the cervical region with well-known fusion of cervical vertebrae 5 and 6 and new constriction of the spinal cord between vertebrae 4 and 5; (**b**) the same shown with three diameters, A, B, and C. The value “Relative Spinal Cord Diameter” (RSD):= 2B/(A+C) can be used in the adapted domain for quantification of the constriction.

However, the process is often more complicated. Therefore, a typical medical example (Figure 6) follows, which requires more in-depth reflection. The comparability of the findings is always necessary, in order to compare experiences (if possible, globally). A female patient had neurological symptoms from the nerves of her cervical spine and an MRI scan was taken of this region. The resulting primary original information (ORGINFO)_1 is a three-dimensional map of the scanned region accessible by the radiologist’s software, which produces images in all planes as secondary original information (ORGINFO)_2. The derived radiology report, ORGINFO_3, contains about half a page of text with an introduction and additional findings. The fusion of cervical vertebrae 5 and 6 was already well-known, but more relevant was the constriction of the spinal cord between vertebrae 4 and 5. In the report, this is described in the following way (translated from German):

“At level C IV/V flat right-sided intervertebral disc prolapse with indentation of the spinal cord...”.

This text (ORGINFO_3) is designed for interpretation by colleagues. It is insufficient, however, concerning precision, comparability, and searchability, as it suffers from the serious problems described in Section 4.2. Therefore, we focus on ORGINFO_2. As part of this focus, Figure 6a shows a relevant cross-sectional image in the sagittal plane. To make such complex findings comparable, according to Section 4.3 and Figure 3, relevant features in this image are searched for. The constriction of the spinal cord between vertebrae 4 and 5 is important and, therefore, quantified. This is possible by calculating the relative diameter of the constricted spinal cord in comparison to the regular diameter. According to Figure 6b, the three lines beside letters A, B, and C represent the diameter of the spinal cord at three locations, where B represents the constricted diameter and (A+C)/2 the non-constricted diameter (as the mean of diameters A and C). Therefore, the value “Relative Spinal Cord Diameter” (RSD):= 2B/(A+C) can be regarded as the quantification of the constriction, which is the relative diameter of the spinal cord at the constricted location. Without constriction, the value of RSD is near to one; otherwise, its value becomes lower with a more constricted spinal cord. Therefore, it is naturally ordered and suitable for a similarity search. Of course, it is not the only interesting parameter. An adapted domain for such findings will contain the date and time, and (in addition to RSD) further interesting dimensions about the patient, such as gender, age, height, weight, physical activity, and so on.

The lengths A, B, and C in Figure 6b should be calculated in a reproducible way by software. Even more precision and validity can be expected from a software-controlled feature extraction of the three-dimensional original map ORGINFO_1. Such a constriction of the spinal cord and of the nerve roots can be quantified by a comparison between the cross-sectional areas. Many parameters can be calculated by such feature extraction. As soon as enough real data are available, those parameters with the best correlation to real clinical findings can be identified to optimize the online definition of the adapted domain. For any interesting topic, the online definition of a meaningfully adapted domain and associated software (e.g., for automatic feature extraction) would be theoretically necessary only once for humankind.

Concerning privacy, it is important that the data of an adapted domain can also be exchanged after averaging, due to their uniform definition. Thus, meaningful medical data can be exchanged globally in an anonymized statistical form [2,3].

## 6. Discussion

### 6.1. Comparison with Current Approaches

There exist well-known resources for standardized communication in medicine, such as ICD (International Statistical Classification of Diseases and Related Health Problems) [12], HL7 (Health Level 7) [8], and SNOMED CT [39]. LOINC (Logical Observation Identifiers Names and Codes) [40] contains many definitions of quantitative data, and every definition has a code. Together with the web address “http://loinc.org”, this code can be used as a globally valid identifier of these data. This is done, for example, using the currently recommended FHIR standard [8,41]. Table 2 shows an excerpt of a “glucose” FHIR example [42].

Lines 05 and 06 of Table 2 contain the LOINC code and address, line 10 contains the date and time, and line 12 contains the value. The unit mmol/L is given in line 13, as well as further additional limit values. As defined in Section 3, this is represented by a UL, plus three numbers in the DV. As the UL addresses the online definition of the subsequent number sequence, it is not necessary to transport more. Units, limit values, and all further details and explanations and cross-references can be integrated uniformly in the online definition. The redundant transport of such data, as shown in Table 2, is unnecessary and can be a source of errors.

More important is the fact that, at present, there is no possibility that users can create online definitions of adapted domains for their topics of expertise (e.g., medical findings), in order to make relevant data in their area of expertise comparable and searchable, as shown in Section 4.3, Section 5.5 and Section 5.6. In this case, the online definition could determine, for instance, that the three following numbers contain the date, time, and the value. These variables need to be transported, and further details are then provided in the online definition (in machine-readable form), which is immediately available to all other users in a uniform way.

### 6.2. User Defined Similarity Search of Medical Information

When comparing data about findings, diagnostics, and treatment, the users (e.g., medical practitioners and specialists) are especially interested in the decision-relevant features; furthermore, they have the best ability to define and quantify such features, due to their knowledge of the subject. After quantification, similarity searching becomes possible. This user-defined similarity search provides an indicator for the comparability of medical information, considering the decision-relevant criteria and, therefore, should be a basal requirement.

At present, however, users are essentially confined to text searches (e.g., literature searches). This is better than nothing, but is hindered by the serious problems described in Section 4.2. Much more precision and reliability are possible in the case of similarity searching in original information; however, at present, this has been restricted to special databases for special applications (e.g., for research). The similarity searching of original medical information for the everyday decisional support of practitioners is not available at present. However, it is important (and is becoming necessary, even) to adequately handle the increasing inundation of multidimensional data. After the selection of the most relevant measurements of a certain patient, a practitioner could search for groups of patients with similar measurements and, within these, for the best treatment decisions. This would be like an individual study and could make it much easier to detect mistakes and to discern successful treatment strategies.

The possibility of similarity searching can be considered as an indicator of the comparability of medical information, as described in Section 4.4. In particular, it is a basal requirement, as a lack of comparability of medical information has far-reaching, everyday consequences. Thus, mistakes are repeated and valuable experiences in medicine are lost. It is, therefore, the responsibility and task of all involved parties (i.e., experts in informatics and medicine) to improve the situation.

From a technical point of view, the FHIR format could also transport certain data for similarity searches (e.g., the quantitative data defined by LOINC, as given in Table 2); however, this is restricted to defined data, coupled with the expense necessary for coding, transporting, and reading these data in forms such as those in Table 2. Such variability impairs comparability.

In contrast, the DV structure is compact, completely defined online, and directly comparable. It can be also used as an extension of FHIR (see Section 3.2, paragraph 5) and its domain can be adapted (Section 4.3) to the situation. For the comparison of medical situations (diagnoses, findings, treatments, and results), adapted domains are necessary to obtain a bijective digital representation of the relevant features in this situation, as explained in Section 4.3 and Section 4.4. Then, reasonable criteria for similarity searches are available. Due to its online definition, the adapted domain is globally defined; therefore, the defined DVs are globally comparable and searchable according to the criteria, which are best defined and updated online by users with the best expertise (e.g., medical specialists; see Section 5.6).

### 6.3. Urgent Questions in Information Science and Informatics

Unsolved and complex interoperability problems have been discussed, but there has been no discussion about the online (i.e., global) definition of information. More than a decade after the publication of [43,44] and long after the publication of [2,3,4] and [45,46], the following questions have become more and more pressing:

1. Why has the exact definition of information as a selection from an ordered set (or domain) (1)not been consequently emphasized and technically utilized from the beginning? This is far-reaching, as adapted domains can be defined online for all possible applications (Figure 3). If it is unclear how to define an ordered set (i.e., domain) and the numbers that select from this set, advanced training (e.g., study of the medical example in Section 5.6) is necessary—information experts (by definition) need to know about this. A “language vocabulary” is only one example of a domain. Semantic concepts and other a posteriori combinations of information are derived applications and also need a basis.
2. Why can users not (especially professionals, experts, and specialists) define adapted domains (Section 4.3) online for precise language-independent global communication in their areas of expertise?
3. Digital information consists of number sequences. Why have these, up to now, been defined in variable and complex ways by context? Why have globally defined, identified, and searchable information carriers (such as the domain vectors detailed above (2), up to now, not been introduced (as selections from an online defined and adapted domain), decades after the introduction of the internet?
4. Why are global information searches still essentially restricted to text searching?

It should be clear that such restrictions have enormous adverse effects (e.g., in medicine). Furthermore, in other professional areas, precise global comparability and precise neutral searches for information would be very advantageous. As preparation for this, the introduction of domain vectors (2), as globally defined searchable information carriers, would be an important step.

Should this basal task not receive support from responsible information scientists?

## 7. Conclusions

The domain of information is crucial for the digital representation of original data. User-guided online definitions of adapted domains for typical medical situations (i.e., diagnoses and treatments) prepare medical information for similarity comparisons, considering decision-relevant criteria and features which are interesting for users.

Therefore, the introduction of domain vectors (DVs, see Section 3), as globally defined searchable information carriers, is recommendable. A first step for this is the establishment of an attractive online presence where users (e.g., medical specialists, experts, and representative patients) can globally, and in a language-independent manner, define adapted domains and domain vectors in their areas of expertise. This allows for a user-defined similarity comparison and medical information searches, which can be integrated into current standards as extensions.

Furthermore, DVs and their definitions can also be used for global interfaces between sub-programs. This allows for the global programming and optimization of modular designs.

## Figures and Tables

**Table 1:** Search results of Figure 5b in detail. The similarity search of DIGINFO = 16 is equivalent to the similarity search of ORGINFO = 16 °C, due to the bijection between ORGINFO and DIGINFO.

ORGINFO	DIGINFO	F = |DIGINFO-16|	RANK
16 °C	16	0	1
15 °C	15	1	2
14 °C	14	2	3
18 °C	18	2	4
0 °C	0	16	5
−5 °C	−5	21	6
45 °C	45	29	7

**Table 2:** Excerpt from the FHIR “Glucose” example [42]. Lines 05 and 06 contain the LOINC web address and the code. Line 10 contains in bold letters the date and time and line 12 contains the value.

Line Code 01 <?xml version="1.0" encoding="UTF-8"?> 02 <Observation xmlns="http://hl7.org/fhir"> 03 <code> 04 <coding> 05 <system value="http://loinc.org"/> 06 <code value="15074-8"/> 07 <display value="Glucose [Moles/volume] in Blood"/> 08 </coding> 09 </code> 10 <issued value="2013-04-03T15:30:10+01:00"/> 11 <valueQuantity> 12 <value value="6.3"/> 13 <unit value="mmol/l"/> 14 <system value="http://unitsofmeasure.org"/> 15 <code value="mmol/L"/> 16 </valueQuantity> 17 <interpretation> 18 <coding> 19 <system value="http://terminology.hl7.org/CodeSystem/ 20 v3-ObservationInterpretation"/> 21 <code value="H"/> 22 <display value="High"/> 23 </coding> 24 </interpretation> 25 <referenceRange> 26 <low> 27 <value value="3.1"/> 28 <unit value="mmol/l"/> 29 <system value="http://unitsofmeasure.org"/> 30 <code value="mmol/L"/> 31 </low> 32 <high> 33 <value value="6.2"/> 34 <unit value="mmol/l"/> 35 <system value="http://unitsofmeasure.org"/> 36 <code value="mmol/L"/> 37 </high> 38 </referenceRange> 39 </Observation>
